# ﻿*Phalaenopsismedogensis* (Orchidaceae, Epidendroideae, Vandeae), a new species from Xizang, China

**DOI:** 10.3897/phytokeys.214.93607

**Published:** 2022-11-22

**Authors:** Chong-Bo Ma, Xi-Long Wang, Wen-Shuai Chen, Zhen Liu, Xiao-Hua Jin

**Affiliations:** 1 State Key Laboratory of Systematic and Evolutionary Botany and Herbarium, Institute of Botany, Chinese Academy of Sciences, Nanxincun 20, Xiangshan, Beijing, 100093, China Institute of Botany, Chinese Academy of Sciences Beijing China; 2 China National Botanical Garden, Beijing 100093, China China National Botanical Garden Beijing China; 3 University of Chinese Academy of Sciences, Beijing, China University of Chinese Academy of Sciences Beijing China; 4 Tibet Plateau Institute of Biology, Lhasa, Xizang 850000, China Tibet Plateau Institute of Biology Xizang China; 5 Motuo Forestry and Grassland Administration, Motuo, Linzhi, Xizang, China Motuo Forestry and Grassland Administration Xizang China

**Keywords:** Flora, Motuo County, orchid taxonomy, Yarlungzangbo River

## Abstract

A new species of Orchidaceae, *Phalaenopsismedogensis*, from Motuo, Xizang, is described and illustrated based on morphological characters and molecular phylogenetics analysis. Molecular phylogenetic analysis and morphological characters indicate that *P.medogensis* is close to *P.deliciosa*, *P.gibbosa* and *P.lobbii*, but differs from them by having triangular wings on the column foot, rhombic lip mid-lobe with a fleshy-horned appendage at the base, and concave lip lateral lobes, the lower part white with a deep purplish-red spot and hairy, the upper part pale yellow with dense rust spots.

## ﻿Introduction

The genus *Phalaenopsis* Bl. comprises 75 accepted names (https://powo.science.kew.org/), distributed from India and Southeast Asia to Australia and New Guinea with centres of diversity in Indonesia and the Philippines (Pridgeon et al. 2014; Deng et al. 2015). Based on molecular and morphological evidence, seven genera, including *Doritis*, *Hygrochilus*, *Kingidium*, *Lesliea*, *Nothodoritis*, *Ornithochilus* and *Sedirea*, have been merged into *Phalaenopsis* s.l. (Tsai et al. 2010; Cribb and Schuiteman 2012; Kocyan and Schuiteman 2014). *Phalaenopsis* s.l. was redefined and a new infrageneric classification was proposed, comprising four subgenera, namely subgen. Parishianae (Sweet) Christenson, subgen. PhalaenopsisBlumesubgen.Hygrochilus (Pfitzer) Kocyan & Schuit, and subgen. Ornithochilus (Lindl.) Kocyan & Schuit (Kocyan and Schuiteman 2014; Pridgeon et al. 2014; Li et al. 2016; Aung and Jin 2021).

There are about 24 species of *Phalaenopsis* in China (Zhou et al. 2021). During our fieldwork in Motuo County, Nyingchi City, Xizang Autonomous Region, China in 2022, an unknown species of *Phalaenopsis* was found in the evergreen broad-leaved forest along the Yarlungzangbo River. Based on morphological characters and molecular evidence, it was identified as a new species of *Phalaenopsis* and is described below.

## ﻿Materials and methods

Morphological characters of the new species were observed, measured and photographed, based on living plants in Motuo, Xizang. Four markers, including one nuclear marker (nrITS) and three plastid markers (*matK*, *trnL* and *trnL-F*), were used in molecular systematics. Primers and amplification procedures of the four markers followed Deng et al. (2015). In total, 40 species in *Phalaenopsis* and seven species of Aeridinae (Suppl. material 1) were included in the molecular analysis. The conflict between nrDNA and plastid DNA data was assessed in PAUA using the length difference test (ILD) (Darlu and Lecointre 2002). Two species, *Aeranthesgrandiflora* Lindley and *Podangisdactyloceras* (Reichenbach) Schltr., were used as the outgroup, based on previous results (Chase et al. 2015; Li et al. 2019). Sequences were edited independently and assembled using SeqMan (https://www.dnastar.com/). Sequence alignment, model selection and super matrix construction were performed in the Phylosuite (Zhang et al. 2020). Bayesian Inference was inferred with MrBayes v. 3.2.7a on XSEDE in the CIPRES Science Gateway online web server (Miller et al. 2010). The model of partition selection was found in ModelFinder (Kalyaanamoorthy et al. 2017) with Corrected Akaike Information Criterion (AICc). GTR+F+I+G4 was selected as the best model for ITS and GTR+F+G4 for the three plastid markers. Two separate Markov Chain Monte Carlo (MCMC) analyses were performed, proceeding for 1,000,000 generations and sampling every 1000 generations. Maximum Likelihood (ML) analyses and model selection were performed in IQ-Tree 2 (Minh et al. 2020). Support values for the clade were estimated using 1,000,000 bootstrap replicates.

## ﻿Results

**Figure 1.**
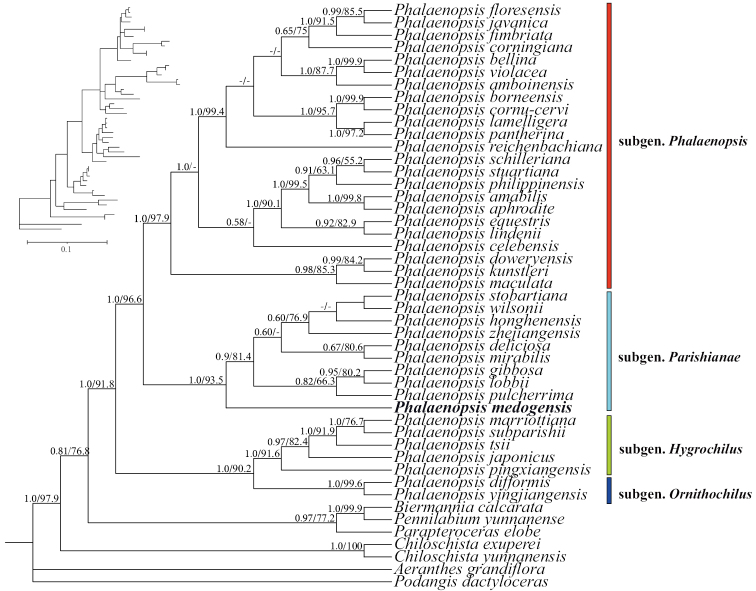
Phylogram of Bayesian Inference based on nrDNA ITS. Numbers above branches indicate posterior probabilities (PP) for BI analysis and bootstrap percentages (BS) for ML, respectively. A dash “-” indicates that support at a node < 50%.

**Figure 2.**
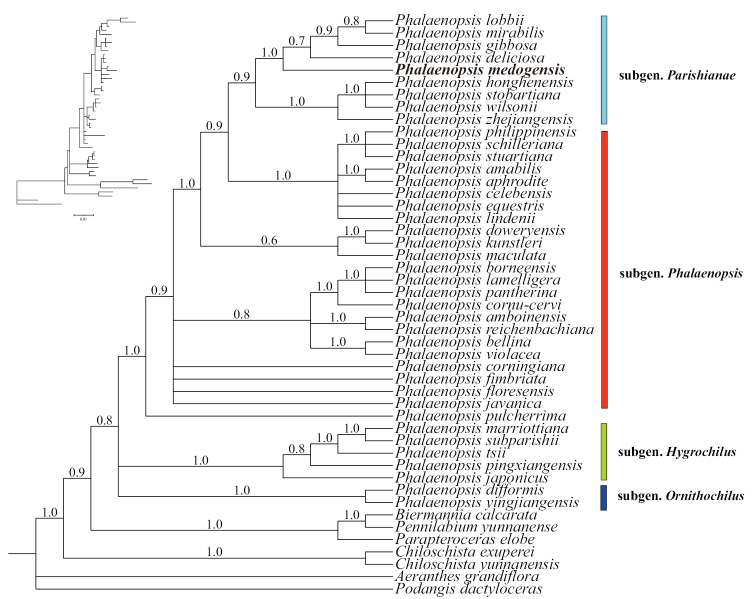
Phylogram of Bayesian Inference, based on plastid sequences (*matK*, *trnL* and *trnL-F*). Numbers above branches indicate posterior probabilities (PP).

The ILD test indicated that plastid markers and nrITS were not suitable for combined analysis. *Phalaenopsismedogensis* belongs to subgen. Parishianae and is close to *P.deliciosa*, *P.gibbosa* and *P.lobbii*, based on molecular phylogenetic analyses. The Bayesian Inference and ML analyses of ITS showed that *P.medogensis* belongs to subgen. Parishianae with moderate to high support (Fig. 1, PP = 1, BS_ML_ = 93.5). The Bayesian Inference of the three plastid markers showed that *P.medogensis* is sister to the group that includes *P.deliciosa*-*P.lobbii* with high support (Fig. 2, PP = 1).

### ﻿Taxonomy

#### ﻿ Phalaenopsis medogensis

Taxon classificationPlantaeAsparagalesOrchidaceae

X.H.Jin & C.B.Ma sp. nov.

F1BBBD5A-3745-5AD3-AC02-8BE758099F62

urn:lsid:ipni.org:names:77308554-1

Figs 3 , 4 墨脱蝴蝶兰

##### Type.

China. Xizang Autonomous Region, Nyingchi City, Motuo County, elev. 800 m, 18 Apr 2022, Xiaohua Jin, Chongbo Ma & Tiankai Zhang 38519 (holotype PE!, isotype, PE!).

##### Diagnosis.

*Phalaenopsismedogensis* is morphologically close to *P.deliciosa*, *P.gibbosa* and *P.lobbii*, but readily distinguished from them by its column foot having a pair of triangular wings, and a lip with concave subrectangular lateral lobes, that are white with a deep purplish-red spot in their lower part and hairy and pale yellow with dense rust-coloured spots in their upper part, and a rhombic mid-lobe with a fleshy horned appendage at base and grooved in the centre (Table 1).

**Figure 3.**
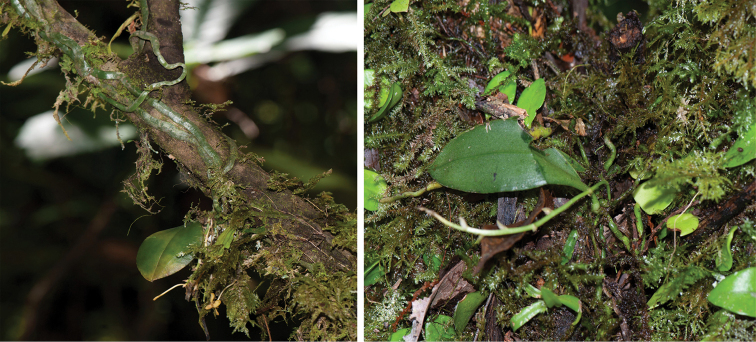
Habitat and plants of *Phalaenopsismedogensis*.

Epiphytic plant. Roots greenish, elongate, flattened, densely verrucose and prostrate along the twigs or trunk. Stem very short. Leaf 1, oblong-elliptic, 7–10 × 3–4 cm. Inflorescences 1 or 2, suberect or arching, ca.12 cm long, unbranched, laxly 5–6 flowered; floral bracts ovate-triangular, 5–6 mm long. Flowers 1–1.2 cm in diameter, yellow; sepals and petals pure yellow, lacking spots or other colouration. Dorsal sepal similar to petals, elliptic, ca. 7–8 × 5 mm; lateral sepals broadly triangular, ca. 10 × 6 mm. Petals long elliptic, ca. 9 × 5 mm. Lip 3-lobed; lateral lobes subrectangular, concave, almost boat-shaped, 2 × 4 mm, lower part white with a deep purplish-red spot and hairy, upper part pale yellow with dense rust-coloured spots; mid-lobe rhombic, ca. 10 × 7 mm, disc grooved, base with a fleshy protuberant appendage; appendage with 2–3 teeth on either side, apex with two long horns. Column subparallel to mid-lobe, ca. 5 mm long; column foot ca. 4 mm long, with triangular wings on both sides; anther cap yellow, hemi-spherical.

**Figure 4.**
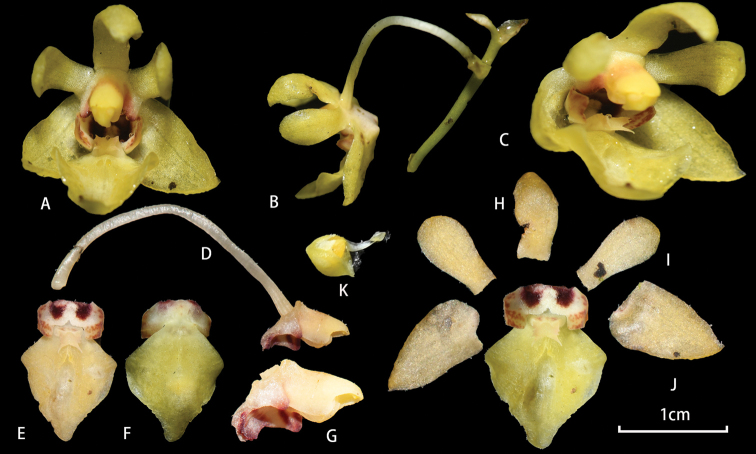
Flowers of *Phalaenopsismedogensis* X.H. Jin & C.B. Ma, sp. nov. **A** front view of flower **B** lateral view of flower **C** front view of flower, showing basal part of lip **D** lateral view of column, ovary and pedicel **E** lip, side view **F** lip, dorsal view **G** column and column foot **H** dorsal sepal **I** petal **J** lateral sepal **K** anther cap. Photographed by Xiaohua Jin and Chongbo Ma.

##### Etymology.

The epithet “*medogensis*” refers to the type locality of the new species, Medog County, Nyingchi City, Xizang Autonomous Region, China.

##### Distribution and habitat.

*Phalaenopsismedogensis* is currently known only from the type locality in Motuo, Xizang, China. It is epiphytic on trunks and twigs at elevations of 700–900m along the hot valley of the Yarlungzangbo River.

##### Phenology.

Flowering in March to April.

##### Conservation status.

*Phalaenopsismedogensis* grows in the tropical rain forest in Motuo County. At least one population of about 30 plants was discovered during our fieldwork. The habitat has been significantly damaged due to the development of agriculture and road construction. We tentatively assessed the risk of extinction of the *Phalaenopsismedogensis* as Critically Endangered (CR) under criteria B2ab(i, ii, iii, iv, v) according to the IUCN criteria version 15.1 (IUCN 2022).

##### Note.

*P.medogensis* is similar to three species in subgen. Parishianae, namely, *P.deliciosa*, *P.gibbosa* and *P.lobbii*, but is readily distinguished from them based on morphological characters given in Table 1.

**Table 1.** Morphological comparison of *Phalaenopsismedogensis* and similar species.

	*P.medogensis*	*P.deliciosa*	*P.gibbosa*	*P.lobbii*
Flower colour	yellow	white with pale purple stripes or markings	white tinged with orange	White
Lateral sepals	broadly triangular, ca. 10 × 6 mm transversely spreading	obliquely ovate, 5.5–6 × 3.5–4 mm curving backwards	obliquely ovate or ovate-elliptic, about 6.5 × 4.5 mm	obliquely ovate to suborbicular, 8 × 7 mm, somewhat reflexed
Mid-lobe of lip	rhombic, 10 × 7 mm; disc grooved with a fleshy protuberant appendage; appendage 2–3 teeth on either side and apex with two long horns	obovate-cuneate, 6 × 5 mm, apex emarginate, with a central ridge; disc with a flattened and Y-shaped appendage	broadly triangular, concave, apex rounded 5–6 × 6–8 mm; disc with a denticulate free callus	reniform, 6 × 10 mm, concave with lateral margins shallowly incurved; disc with a callus of four filiform appendages
Lateral lobes of lip	subrectangular, concave, almost boat-shaped, lower part white with a deep purplish-red spot and hairy, upper part pale yellow with dense rust-coloured spots	obliquely elliptic-obovate, rounded, with tooth-like flaps, base decurrent and forming a broadly conic spur	erect, linear, falcate, acuminate	erect, falcate, parallel to middle and then diverging and forming a U-shaped structure
Column foot	with triangular wings on both sides	without wings	without wings	without wings

### ﻿Key to species of subgen. Parishianae in China

**Table:** 

1a	Leaves deciduous; lip mid-lobe smaller than petals and sepals and with central protuberance	**2**
1b	Leaves more or less persistent; lip mid-lobe broad, lacking an obvious protuberance or concave U-shaped compound structure in centre	**5**
2a	Column obviously elongated, ca. 5 × 1.2 mm, near base with an appendage; rostellum apex hooked and slightly bilobed, petals wider than sepals	***P.zhejiangensis***
2b	Column not obviously elongated, without an appendage at base; petals and sepals similar in width	**3**
3a	Lip mid-lobe obcordate with a central apical fleshy knob	***P.wilsonii***
3b	Lip mid-lobe of lip not obcordate, lacking a terminal notch	**4**
4a	Flowers pink; lip mid-lobe with a conspicuous constriction	***P.honghenensis***
4b	Flowers deep green; lip mid-lobe oblanceolate, lacking any conspicuous constriction	***P.stobartiana***
5a	Terrestrial or lithophytic; lip mid-lobe split into three lobelets	***P.pulcherrima***
5b	Epiphytic; lip mid-lobe entire, slightly concave	**6**
6a	Column base not protuberant; lip more or less spurred; lateral lobe margins dentate	**7**
6b	Column base distinctly protuberant; lip not spurred; lateral lobes entire	**8**
7a	Lip mid-lobe sagittate; spur sacculate	***P.mirabilis***
7b	Lip mid-lobe obovate-cuneate, apex deeply emarginate, with a thickened central longitudinal ridge; spur indistinct	***P.deliciosa***
8a	Lip mid-lobe rhombic, its base with a fleshy protuberant appendage; appendage with 2–3 teeth on either side, apex with two long horns; column foot with triangular wings on both sides	***P.medogensis***
8b	Lip mid-lobe non-rhombic, its base with a callus with filiform appendages	**9**
9a	Lip mid-lobe white with one broad longitudinal chestnut-brown stripe, the basal callus deeply forked, with a crescent-shaped appendage in middle, each arm of the callus divided into two filiform-linear antennae	***P.malipoensis***
9b	Lip mid-lobe white with two broad longitudinal chestnut-brown or yellow stripes, its basal callus of four filiform appendages	**10**
10a	Lateral sepals transversely spreading, ovate-elliptic	***P.lobbii***
10b	Lateral sepals curving backwards, ovate	***P.gibbosa***

## Supplementary Material

XML Treatment for Phalaenopsis medogensis

## Supplementary materials

Supplementary material 1 Voucher and GenBank accession numbers Data type Docx file. https://binary.pensoft.net/file/772640This dataset is made available under the Open Database License (http://opendatacommons.org/licenses/odbl/1.0/). The Open Database License (ODbL) is a license agreement intended to allow users to freely share, modify, and use this Dataset while maintaining this same freedom for others, provided that the original source and author(s) are credited.Chong-Bo Ma Wang Xi-Long, ChenWen-Shuai, Liu Zheng, Xiao-Hua Jin
