# Supplementary material for: ﻿Phalaenopsismedogensis (Orchidaceae, Epidendroideae, Vandeae), a new species from Xizang, China
Source: PhytoKeys. 2022 Nov 22;214:39–46. doi: 10.3897/phytokeys.214.93607 (PMC9836467; doi:10.3897/phytokeys.214.93607)
Supplement: Supplementary material 1 — Voucher and GenBank accession numbers [file phytokeys-214-039_article-93607__-s001.docx]

**Table S1.** Voucher and GenBank accession numbers.

| Species | Voucher | ITS | trnL intron | trnL-F spacer | matK |
| --- | --- | --- | --- | --- | --- |
| *Aeranthes grandiflora* | Liu 7178 | KJ021017 | KJ021009 | KJ021009 | KJ021021 |
| *Biermannia calcarata* | Liu 6144 | KJ733388 | KJ733625 | KJ733625 | KJ733547 |
| *Chiloschista exuperei* | Liu 4976 | KJ021016 | KJ021008 | KJ021008 | KJ021020 |
| *Chiloschista yunnanensis* | Liu 6094 | KJ021015 | KJ021007 | KJ021007 | KJ021019 |
| *Parapteroceras elobe* | Liu 4596 | KJ733427 | KJ733664 | KJ733664 | KJ733584 |
| *Pennilabium yunnanense* | Liu 5471 | KJ733431 | KJ733668 | KJ733668 | KJ733588 |
| *Phalaenopsis amabilis* |  | AY391524 | AY265742 | AF533472 | EU256323 |
| *Phalaenopsis amboinensis* |  | AY912217 | AY265743 | AF533458 | AY498548 |
| *Phalaenopsis aphrodite* |  | AY391537 | AY265744 | AF533473 | AY498549 |
| *Phalaenopsis bellina* |  | AY900290 | AY265746 | AF533467 | AY513768 |
| *Phalaenopsis borneensis* |  | AF537024 | AY265747 | AF533476 |  |
| *Phalaenopsis celebensis* |  | AF537014 | AY265799 | AF533466 |  |
| *Phalaenopsis corningiana* |  | AY390247 | AY265750 | AF533448 |  |
| *Phalaenopsis cornu-cervi* |  | AY912222 | AY265751 | KC985424 | KC823038 |
| *Phalaenopsis deliciosa* |  | KJ733433 | KJ733670 | KJ733670 | KJ733590 |
| *Phalaenopsis difformis* | Liu 7160 | KF545878 | KF545900 | KF545900 | KF545889 |
| *Phalaenopsis doweryensis* |  | AY912224 | AY265753 | AF533485 |  |
| *Phalaenopsis equestris* |  | AY912225 | AY265754 | AF533464 | AF263677 |
| *Phalaenopsis fimbriata* |  | AF537013 | AY265756 | AF533465 |  |
| *Phalaenopsis floresensis* |  | AY912227 | AY265797 | AF533462 | MT518651 |
| *Phalaenopsis gibbosa* |  | AY912228 | AY265758 | AF533461 |  |
| *Phalaenopsis honghenensis* |  | AY912229 | DQ195040 | DQ194995 |  |
| *Phalaenopsis japonica* | Liu 2493 | KF545880 | KF545902 | KF545902 | KF545890 |
| *Phalaenopsis javanica* |  | AY912231 | AY265763 | AF533455 |  |
| *Phalaenopsis kunstleri* |  | AY912232 | AY265764 | AF533486 |  |
| *Phalaenopsis lamelligera* |  | AY912233 | AY265765 | AF533477 | EU179845 |
| *Phalaenopsis lindenii* |  | AY912234 | AY265766 | AF533480 |  |
| *Phalaenopsis lobbii* |  | AY912235 | AY265767 | AF533474 |  |
| *Phalaenopsis maculata* |  | AF537008 | AY265798 | AF533460 | MT518657 |
| *Phalaenopsis marriottiana* | Liu 3336 | KF545876 | KF545898 | KF545898 | KF545887 |
| *Phalaenopsis mirabilis* |  | AB217559 |  |  | AB217735 |
| *Phalaenopsis pantherina* |  | AY912244 | AY265775 | AF533463 |  |
| *Phalaenopsis parishii* |  | AY912245 | AY265774 | AF533491 |  |
| *Phalaenopsis philippinensis* |  | AY912246 | AY265776 | AF533446 |  |
| *Phalaenopsis pingxiangensis* | Liu 6651 | KX579761 | KX579767 | KX579767 | KX579763 |
| *Phalaenopsis pulcherrima* |  | AY912247 | AY265777 | AF533495 | MT518661 |
| *Phalaenopsis reichenbachiana* |  | AY912249 | AY265779 | AY266120 | MT518662 |
| *Phalaenopsis schilleriana* |  | AY912250 | AY265781 | AF533443 |  |
| *Phalaenopsis stobartiana* |  | KP976020 | KP976028 | KP976036 |  |
| *Phalaenopsis stuartiana* |  | AY912251 | AY265782 | AF533492 |  |
| *Phalaenopsis subparishii* | Liu 6077 | KF545881 | KF545903 | KF545903 | KF545891 |
| *Phalaenopsis tsii* | Liu 7149 | KF545877 | KF545899 | KF545899 | KF545888 |
| *Phalaenopsis violacea* |  | AY390229 | AY265796 | AF533487 | MT518666 |
| *Phalaenopsis wilsonii* |  | AY912257 | AY265787 | AF533475 | AB217751 |
| *Phalaenopsis yingjiangensis* |  | KF545879 | KF545901 | KF545901 | KF545894 |
| *Phalaenopsis zhejiangensis* |  | KF545873 | KF545895 | KF545895 | KF545884 |
| *Podangis dactyloceras* | Liu 7179 | KJ021018 | KJ021010 | KJ021010 | KJ021022 |
| *Phalaenopsis yarlungzangboensis* | Jin et al. 38519(PE) | ON677963 | ON691649 | ON691649 | ON691648 |
